# How consequences of colorectal cancer treatment are managed: a qualitative study of stakeholder experiences about supportive care and current practices

**DOI:** 10.1007/s00520-023-07713-7

**Published:** 2023-04-11

**Authors:** Claudia Rutherford, Angela Ju, Bora Kim, Lisette Wiltink, Louise Acret, Kate White

**Affiliations:** 1 Cancer Care Research Unit (CCRU), Susan Wakil School of Nursing, University of Sydney, Sydney, Australia; 2 University of Sydney, Faculty Science, School of Psychology, Quality of Life Office, Sydney, Australia; 3 The Daffodil Centre, The University of Sydney, a joint venture with Cancer Council NSW, Sydney, Australia; 4 Department of Radiation Oncology, Leiden University Medical Centre, Leiden, the Netherlands; 5 Sydney Local Health District, Sydney, Australia

**Keywords:** Bowel cancer, Qualitative study, Symptoms, Treatment effects, Survivorship

## Abstract

**Purpose:**

Colorectal cancer (CRC) survivors experience treatment-effects such as symptoms and functional impairments. There is limited evidence about how these are managed and what services or supports are available in the community. We aimed to identify current practice and available supports for managing consequences of treatment from clinician and CRC survivor perspectives.

**Methods:**

This qualitative study, informed by an interpretivist constructionist paradigm, included semi-structured interviews. Clinicians with experience of treating CRC patients and adult CRC survivors were recruited across Australia. Interviews explored experiences about problems experienced after CRC treatment and how these were managed. Data collection and analysis, using thematic analysis, was iterative whereby emergent themes during analysis were incorporated into subsequent interviews.

**Results:**

We interviewed 16 clinicians and 18 survivors. Survivors experienced a range of consequences of treatment amendable to support including allied health, information, and self-management. Barriers to support access included clinicians’ worry about patient out-of-pocket expenses, long waitlists, lack of awareness about existing supports, and perception no therapeutic options were available. Healthcare professionals with expertise in CRC were often difficult to identify outside of cancer settings. Survivorship care could be improved with individualised timely information and identification of pathways to access healthcare providers with expertise in managing consequences of CRC treatment within primary care.

**Conclusions:**

To improve CRC survivor lives posttreatment, routine assessment of consequences of treatment, individualised care planning involving relevant healthcare professionals, access to supportive care when needed, and improved information provision and engagement of a range of health professionals in follow-up care are needed.

## Background

Colorectal cancer (CRC), including bowel, colon, and rectal cancer, is an increasing malignancy in the developed world [1]. In 2018, 1.8 million new cases were recorded and almost 861,000 deaths [1]. Despite the high incidence, survival rates are improving, leading to a growing population of CRC survivors. The overall 5-year survival rate is 64–69% but can be as high as 90% if diagnosed at early stage [2, 3].

Longer life years gained can be attributed to advances in screening, early diagnosis and effective treatments. However, CRC and its treatments can affect patients in many ways [4, 5], causing symptoms, side-effects, and functional impairment, all of which can impact health-related quality of life (HRQL) during treatment and in posttreatment survivorship [6, 7]. Further, CRC survivors experience persistent symptoms such as faecal incontinence, frequency and pain, and functioning impairments long after treatment completion [6, 7] and report poorer physical function, depression, and HRQL than the general population [8, 9]. Many of these problems remain unmanaged and about half of CRC survivors report unmet needs related to sexual dysfunction, fatigue, pain, and bowel control [10, 11]. At 2-years posttreatment, about one-quarter experience at least one moderate or severe unmet need [11]. Psychological and physical unmet needs are the most common and do not improve over time. Evidence from international studies suggests other long-term effects such as short bowel syndrome, faecal urgency, and altered body image and sexual function [12].

Clinicians such as oncologists, nurses, and general practitioners involved in the care of patients with CRC play an important role in addressing their unmet needs. However, current clinical practice around referrals and provision of health services and management options for gastrointestinal symptoms and functioning impairments remains unclear. Clinicians may be unaware of targeted interventions aimed at detecting and managing posttreatment effects and survivors of CRC report self-managing their gastrointestinal symptoms and functioning impairments rather than seeking professional help [6]. What is unclear is why this is: is it because appropriate health services or supports do not exist or that they do exist, but patients and clinicians do not know they exist or how to access them. The potential for early intervention to detect and ameliorate consequences of treatment is significant. This study aimed to identify current practice for managing consequences of treatment in CRC survivors and experiences about available supports for managing these from both clinicians and survivors of CRC perspectives. “Supports” from here on refers to any health service or health professional that provides specific intervention, both specialist and primary care-based. This includes, but not limited to, pelvic floor physiotherapists, stomal therapy nurses, specialist continence nurses, sexual therapists, and psychologists and is inclusive of the specific interventions or management strategies provided by these providers.

## Methods

### Study participants

This qualitative study included clinicians with experience of treating patients with CRC, including nurses, CRC surgeons, radiation oncologists, medical oncologists, gastroenterologists, dieticians, general practitioners, social workers, and psychologists; and cancer survivors with current or previous experience of CRC if they fulfilled the following criteria:

-  ≥ 12 months since diagnosis
- Aged ≥ 18 years
- Able to share their experience in English
- Able to give written informed consent to take part

The study was informed by an interpretivist constructionist paradigm [13], allowing for detailed investigation both within and across interviews and comparison between participant groups.

### Participant recruitment and consent procedures

To recruit a broad sample of clinicians and CRC survivors, we disseminated a study invitation widely, with a detailed participant information statement. It was disseminated through our collegial networks, international cancer institutes and professional societies, cancer patient support groups, and Twitter. We also utilised a snow-ball recruitment strategy where upon participation, each participant was asked to invite their peers to the study by forwarding them the study invitation. Those interested in taking part were asked to email or telephone the researcher directly. The researcher provided further details about the study and arranged a time and date for the interview. A consent form for the interview was sent in an email confirming the time and date for the interview for all participants who agreed to take part. All participants who took part in the interview provided either written or recorded verbal consent. Study recruitment took place between 24 October 2019 and 06 June 2020. The study was reviewed and approved by the University of Sydney Human Research Ethics Committee (HREC) (Project No. 2019/714).

### Data collection

Semi-structured interviews were conducted by an investigator trained in qualitative research methods and guided by an interview schedule (Box 1). Interviews were conducted via telephone or Zoom, depending on participant preference, and audio-recorded. Recruitment ceased when data saturation was met (i.e. when no new information was being generated). Accrual was reviewed to ensure balanced representation of different clinical professions and patient characteristics. Interviews explored in-depth experiences about commonly experienced consequences of treatment for CRC and how these were managed (see Box 1 for detailed topics covered in interviews).

**Box 1:** Outline of clinician and CRC survivor interview script

*Clinicians*
1) Common problems reported by CRC survivors after treatment and how clinicians currently managed these
2) Currently available interventions or referral pathways for managing gastrointestinal symptoms and functioning impairments in CRC survivors
3) Experiences about intervention effectiveness and needs for new or improved interventions
*CRC survivors*
1) How survivors currently managed their gastrointestinal symptoms and functioning impairments
2) Interventions tried and experiences of what worked/didn’t work
3) Other interventions they were aware of but not tried
4) Needs for new or improved interventions

## Data analysis

Data collection and analysis involved an iterative process whereby emergent issues identified during analysis were incorporated into subsequent interviews [14]. Emergent themes were identified using a thematic analysis framework [14, 15]. Data collected was assigned conceptual labels. This involved breaking down the data into discrete “findings” and coding related “findings” into descriptive themes [14]. For example, findings that “established clinical pathways”, “streamlined referrals” and “improved availability and access” reflect similar phenomena and were coded to a “Better access to existing interventions” theme. Our analysis developed in an inductive manner, whereby themes were developed through constant comparison of the similarities and differences in the findings, searching for both supportive and disconfirming evidence. The preliminary themes identified by one researcher were reviewed independently by a second to ensure that the themes captured the depth and range of data collected across interviews. Discrepancies were discussed and revisions to the themes agreed.

## Results

We interviewed 34 participants: 16 clinicians (Table 1) and 18 survivors (Table 2).

**Table 1:** Demographic characteristics of clinicians (*n* = 16)

Variable	*n*
Gender	
Male	4
Female	12
Primary clinical specialty	
Advanced nurse practitioner	4
Stoma nurse	2
Surgeon	3
Medical oncologist	2
Radiation oncologist	1
Psychologist	1
Dietician	2
GP	1
Healthcare setting	
Private	1
Public	10
Mixed	5
Geographical location	
Metropolitan	13
Rural	3
Number of years post-qualification	
< 1	0
1–5	1
6–10	3
> 10	12
Number of CRC patients seen each year	
< 10	1
10–50	3
51–100	2
> 100	10

**Table 2:** Demographic characteristics of survivors (*n* = 18)

Variable	*n*
Gender	
Male	11
Female	7
Age	
31–40	1
41–50	0
51–60	3
61–70	8
71–80	4
80 and above	2
Geographical location	
Metropolitan	15
Rural	3
Length of time after initial diagnosis	
1–2 years	1
3–4 years	2
5–9 years	11
10 or more years	4
Primary cancer diagnosis	
Colon	15
Rectum	3
Presence of stoma	
Yes	3
No	8
Yes but removed/reversed	7
Treatment type (1 or more)*	
Surgery	18
Chemotherapy	14
Radiotherapy	4
Metastasis	
Yes	6
No	11
Unsure	1

### Clinician experiences

#### Consequences of treatment

Consequences of treatment, including symptoms and side effects, depended on cancer and treatment type, but the most common were bowel changes, fatigue, pain, sexual concerns, stress and anxiety. Bowel cancer patients commonly reported anaemia and rectal cancer patients altered bowel habits and rectal bleeding. Pain, weight loss, inability to eat, and symptoms of obstruction were most commonly associated with recurrence and sciatic pain and fatigue with pelvic exenteration.

#### Assessment of consequences of treatment

Most clinicians reported doing no formal assessment of posttreatment effects. When done, assessment was usually verbal without a checklist or reliance on patient self-reports. Particularly surgeons discussed only the issues patients “brought up”. Few used established tools (Box 2). Clinicians felt routine screening for known possible treatment effects and unmet needs were needed. Patient-reported outcome measures might be useful for this purpose as part of a team-based follow-up approach but needed integration with the cancer record and appropriate thresholds established for intervention pathways.

#### Specialist support to manage consequences of treatment

Referrals were made, mostly by surgeons, to a range of healthcare professionals (Box 2). Clinicians found survivorship clinics, anorectal clinics, urology sexual dysfunction clinics, CRC nurses, stoma therapists, pelvic floor specialists, and GPs most helpful for managing specific patient needs. Where possible, telehealth was a valuable option for unwell or remote patients.

**Box 2:** Available supports

Patient-reported questionnaires used by clinicians to assess consequences of treatment
• Validated tool used by surgeons to assess bowel function
• Victoria unmet needs tool
• Supportive care screening tool (“very good but not used much in practice”)
• Patient generated subjective global assessment
• QLQ-C30 and QLQ-CR29 (one surgeon asked their patients to complete these questionnaires prior to their clinical visit)
• Schofield equation (dieticians used to calculate energy requirements)
**Specialist referrals**
Most commonly, patients were referred to stoma specialists or dieticians for bowel problems, psychologists for psychological or sexual issues, or prescribed analgesics for pain. Less commonly, patients were referred to physiotherapists, dieticians, stoma therapists, pain specialists, palliative care, medical and radiation oncologists, psychologists, and urologists
**Written information for patients**
• Pamphlets on “Bowel Cancer: The questions you need to ask”, “Bowel Cancer: What follow-up should I have?”
• Australian Government booklet “Improving Bowel Function After Surgery”
• Evi-Q chemotherapy patient information hand-out
• Written information about supplements
**Interventions offered to patients to manage consequences of treatment**
• Loperamide
• Diet changes (e.g. avoid irritating foods, have smaller regular meals throughout the day)
• Transanal irrigation
• Sacral nerve stimulation
• Biofeedback
• Blended therapy (e.g. distress screening then targeted counselling based on a patients’ symptom profile)

#### Information provided by clinicians

Clinicians supported CRC survivors by providing information about lifestyle changes, toileting techniques, skincare, hygiene, exercise, and diet in the form of readily available printed or online materials (Box 2). These were supplemented by a discussion with members of the multidisciplinary team, who explained and elaborated on important points or those on which patients needed clarity. Surgeons routinely provided information about required follow-up and how to reduce the risk of recurrence through PowerPoint presentations, written booklets/pamphlets, and links to online materials from professional bodies. Less was covered concerning supportive care and management of treatment effects. Consistently clinicians felt there were too many different information resources, both printed and online, which could be overwhelming for patients and difficult for clinicians to know which to recommend.

#### Barriers to support access and/or referral to services

##### Costs of supportive care interventions

Clinicians’ concerns over patient costs associated with specialist services were a barrier to referral. They felt there were limited government-funded health services for ongoing support after hospital discharge so clinicians did not routinely refer patients. Others felt certain services (e.g. pelvic floor physiotherapist) were not available locally, or had long wait-lists (e.g. sexual function clinics), which prevented access or incurred additional costs.

##### Clinicians’ awareness of available supports and perspective about referral

Clinician’s acknowledged needing better awareness of available supports to enable appropriate referrals and provision of holistic care. One example given was “lack of awareness about government-funded supply of pads for incontinence to eligible patients”. Clinicians used their clinical experience rather than a standardised approach to who was offered supports. Some felt they lacked knowledge and competency to recommend appropriate supports, while others perceived this beyond their scope of practice or outside their area of expertise. Others did not start conversations about supportive care needs due to time constraints or personal discomfort.

I feel like a tape recorder asking everyone about sexual and urinary and bowel function…sometimes I just gloss over that stuff and hope that by the time rapport has been established, [the patient] will volunteer that information. – female, surgeon.

#### Facilitators to support access and/or referral to specialist services

##### Improved access to existing supports

Clinicians acknowledged that supports were likely available, but raising awareness of these as well as establishing care pathways or automatic referrals to specialists (e.g. cancer nurses, physiotherapists, psychologists, dieticians) with understanding of CRC specifically was needed to facilitate better access. Free or affordable dieticians and pelvic floor specialists to help manage bowel function after CRC treatment was desirable. Partnering with industry to improve appliances and technologies, representation on government committees to leverage funding for the stoma pouch scheme, and Medicare support for cancer nurse navigators, nurse coordinators, or stomal therapy nurses to coordinate care after discharge from hospital was suggested.

##### Multidisciplinary approach to supportive care

Clinicians consistently reported the need for better inclusion of various healthcare providers in preoperative MDT meetings, improved understanding of posttreatment issues important to CRC survivors, and access to services and supports to manage them, particularly bowel and sexual function issues. They felt these issues were inconsistently managed, and information about or referral to appropriate supports was ad hoc. Survivorship clinics or programmes, intimacy clinics, and better access to psychologists and stomal therapists would be beneficial in both hospital and community settings.

We’ve become more proactive about asking about bowel function with … scores and recognising it early and referring. But that’s in big centres, I doubt they are in smaller ones – female, surgeon

### Gaps in supports for CRC survivors

Several gaps were discussed. First, better ways to detect ongoing problems were needed, as currently it was up to clinicians to ask about all possible treatment effects. A follow-up call post-discharge from hospital would be beneficial to discuss any ongoing problems or new issues that arose posttreatment. Second, there was need for better access to existing supports to address common problems like urinary and faecal dysfunction, sexual health, and diet. Services were available, some of which were provided in cancer centres such as specialist nurses and physiotherapists but required patients to return to the acute setting. Allied health specialists and services located in the primary care setting were less known and may require financial payment and referral pathways. Further, knowledge of their existence was problematic and outside of cancer, and the gaps were in the mechanism by which they could be accessed. Tapping into existing supports, especially those based in the community, would be more sustainable than creating new roles located within specialist cancer centres. A register of healthcare providers’ specialist knowledge/expertise in CRC (e.g. psychologist with expertise of cancer survivorship; physiotherapist with expertise in pelvic floor problems) would be a valuable resource to both clinicians and patients. Third, education options for healthcare providers about CRC-treatment effects and how to implement optimal care pathways outside of cancer centres may be of interest to some clinicians. More research and development into supports for bowel and sexual function, anal muscle damage, neuropathy, fatigue, and disturbed sleep were also needed. Finally, there was need for supports specifically for young survivors of CRC, especially education about sexual function, relationships, and fertility, but these should occur at diagnosis. A phone line for patients to call about specific problems and better stoma support, such as a stoma buddy programme, was thought could also be beneficial.

### Experiences of survivors of CRC

#### Survivors posttreatment experience

Survivors experienced various symptoms and functioning impairments posttreatment. Some were short-term after completing chemotherapy or surgery, such as nausea and wound pain. Others were long-lasting such as neuropathy, chronic pain, altered bowel habits and taste (which affected appetite and weight), fatigue, poor concentration, and skin reactions.

It felt like I never recovered from [surgery], both from ongoing pain and the fatigue was crippling… I hit the wall by 8pm every night. I did a volunteer shift one day a week which was all the work I could manage, and I would be shaking and shivering with exhaustion all the way home afterwards – female, survivor

Non-physical concerns such as difficulties returning to work, financial hardship, and emotional difficulties such as fear of recurrence and anxiety were frequently reported. Those with a stoma pouch experienced burning, bleeding, leakages, and blockages. Learning to manage a stoma pouch was challenging and overwhelming as it was difficult to know what was normal versus concerning.

Most people with cancer understand they have to go through the stress and anxiety about recurrence every time you have a routine scan – male, survivor

Feeling like treatment “would never end”, fear of recurrence, peripheral neuropathy, ongoing fatigue, and poor concentration/memory had the greatest impact on individuals’ lives. Many self-sought ways to manage posttreatment effects, including doing their own research, talking with others, and changing day-to-day routines to manage physical symptoms. Support groups provided peer support and an avenue to hear about others’ first-hand experiences.

Hard to say [what interventions are needed], I don’t feel like anybody can do anything for me… it’s up to me – male, survivor

Surgeon and medical staff were excellent with the clinical stuff, not so much help for the emotional [issues] – male, survivor

#### Inadequate information provision and support access

Most participants had not expected the posttreatment effects they experienced, feeling ill-prepared, “lost”, and “alone”, and not knowing who to contact for support. This was particularly troubling for people in rural areas with poorer access to supports. Several participants received written materials, some of which were considered helpful. Others sought help from their GPs. Although some had positive experiences with their GPs, most reported their GP played a minimal role in managing posttreatment effects. Some GPs were proactive in asking about a range of issues, but many lacked specific knowledge relating to CRC treatment effects such as neuropathy. When received, support from stoma therapists after completing treatment was extremely helpful.

No information about what to expect 6 months – 2 years… stoma nurse marked where the stoma will go but didn’t tell [me]… surgeon went through risks of surgery than what to expect long term… oncologist didn’t say much about symptoms either. Only 6 months after when the stoma bag came off, symptoms come through but I wasn’t aware [of what to expect] – male, survivor

I didn’t realise [recuperating from surgery] was going to be this bad, changed my life that much – female, survivor

#### Survivor perceived barriers to seeking support

Barriers to seeking support for managing CRC-treatment effects included worry about financial security, resulting in many choosing self-management or complementary medicine options. Symptoms were perceived as “very personal” and survivors felt embarrassed to raise certain issues. Some survivors did not want to ask for help, only to be told “it’s normal and you just need to clean it up”. Others noted that “western medicine is about helping you but they also want you to take care of yourself”. Survivors also worried raising issues would result in more tests or be perceived as a “bad treatment outcome” by their cancer specialist. Not knowing who to speak to when posttreatment effects occurred was another barrier. Finally, GPs were perceived to have insufficient expertise to solely deal with post-CRC treatment needs.

I was reluctant to tell the surgeon about certain problems because I worried it would end up in more treatment or perceived as a bad outcome of the [primary] treatment – female, survivor

### Survivor perceptions on ways to improve supportive care

#### Individualised and timely information provision

Providing the “right individualised information at the right time” was considered essential. Patients had limited capacity for processing information during treatment, so repeating patient education after treatment was necessary. Participants desired more information about the management of their stoma pouch, statistics around the likelihood of experiencing certain symptoms/side effects, and interventions to manage anxiety, fear of recurrence, physical symptoms, and return to work matters.

Cancer nurses [after the initial diagnosis] are very helpful in consolidating the immense amount of information out there – male, survivor

#### Available and accessible supports in the community

Importance of having access to a range of healthcare providers with expertise in managing CRC-treatment effects was needed. For example, a psychologist with an understanding of cancer-related emotional impacts could offer context-specific strategies. Likewise, a physiotherapist or dietician with knowledge in CRC could provide specific advice on diet and exercise to promote functional recovery. However, better ways to advertise and promote healthcare providers with expertise in CRC were needed, which would enable GPs to play a greater role in coordinating posttreatment care and linking people with appropriate supports in the community.

Someone who [patients] can call for support, check if symptoms are normal or needs attention… GPs may not always be available without an appointment – female, survivor

Public patients have a huge problem because of the waiting list for immediate treatment and supports – male, survivor

#### Central care coordinator

Several participants emphasised the importance of a designated care coordinator, particularly in the community and especially in rural and regional areas. A central person of contact with in depth knowledge of the patient and their disease and treatment would be a key source for coordinating care after treatment. CRC was perceived as a highly specialised area requiring specialists such as dieticians and pelvic floor therapists, not just cancer specialists. Development of individual care plans and a central telephone or online chat service that linked patients with local supports based on their specific needs was required.

[We need] one person overall coordinating, rather than different specialties – female, survivor

### Comparison of survivors and clinicians

Direct consequences of treatment for CRC such as bowel changes and pain were noted by both clinicians and survivors. However, survivors noted additional concerns such as financial impact and poor concentration following their primary treatment. While clinicians routinely made referrals and provided information for perceived or raised issues, survivors reported feeling isolated and unequipped to manage their consequences of treatment. Clinicians were wary of overwhelming their patients and reported feeling unsure of what information or referrals would be most helpful for them. Clinicians perceived cost and availability locally as the biggest barriers to CRC survivors accessing specialist services. On the other hand, CRC survivors reported several barriers to contacting their clinicians for help, such as uncertainty around who to contact and fear of being told to “just deal with it”. Resolving some of these discrepancies in perceptions between clinicians and survivors may help to address the unmet needs of survivors of CRC.

## Discussion

Survivors of CRC experienced a range of consequences of treatment that were short term to life long. Many felt ill-prepared for these consequences when discharged from hospital and often those they experienced were unexpected. Formal assessment of posttreatment consequences by clinicians was rare and ad hoc and often left up to patients to bring up. Clinicians identified that they were the greatest barrier to patients accessing supports due to not asking about support needs, not knowing about available services or how to access them, concern over patient out-of-pocket expenses, and thinking certain options were not available locally or had long waitlists. Clinicians acknowledged that supports for managing consequences of CRC-treatment were likely available, and reviews identify interventions for psychological [16], physical [17], and sexual [18] concerns, amongst others. However, clinicians were unclear of their role in assessing consequences of treatment and referring patients to other services or supports. The problem lies in part with poorly defined roles and responsibilities and no professional designated as “coordinator of survivorship care”. Further compounding the problem is that healthcare professionals do not always promote themselves as having expertise in CRC. Clinician barriers led to them acting as gatekeepers to referral and access. Gatekeeping has been associated with lower healthcare use and expenditure and better quality of care, but lower patient satisfaction and cancer survival rates, although primary care gatekeeping was not associated with delayed patient referral [19]. Participants suggested that methods to detect consequences of treatment, established care pathways, automatic referrals to allied health professionals with an understanding of CRC, development of survivorship care plans, and a central telephone service that linked patients with local supports based on their specific needs would facilitate better access.

When discussing support options, clinicians mainly referred their CRC patients to other specialists such as dieticians and stoma therapists. Few discussed self-management or behaviour change interventions with their patients. Survivors did not find any one support effective for any specific consequence of treatment. Rather, one support worked for one person but not another. Some trial-and-error seems inevitable while finding what works for an individual. Thus, informing patients and setting realistic expectations may reduce some worry experienced when consequences of treatment occur. Survivors consistently felt that supportive care could be improved with the right information at the right time, access to a range of healthcare providers with expertise in managing consequences of CRC-treatment, and a designated coordinator of care posttreatment. Patients with fulfilled information needs and who experience less information barriers, in general, have better quality of life and less anxiety and depression [20].

Participants identified gaps in supports. Interventions for bowel and sexual function, anal muscle damage, neuropathy, fatigue, disturbed sleep, and fear of cancer recurrence are needed. A range of healthcare professionals are required to manage consequences of CRC-treatment and meet the needs of survivors in posttreatment survivorship. Many are available in primary care but the mechanism by which they can be accessed needs improving. When a range of healthcare professionals have been included in preoperative MDTs, particularly when they all inform care planning [21], patients received adequate support with managing symptoms and treatment effects, adequate nutritional and psychological support, and individualised care planning during posttreatment follow-up [21–23]. When survivorship care plans have been used, particularly plans which set out actions to be taken to meet individual needs [24], they were associated with survivor perceived timely support that met their informational, physical, emotional, and practical needs posttreatment [25].

This study has some limitations. Participants opted in to participate in this study so they may be individuals interested in this topic. Our sample included only one participant aged between 31 and 40. Younger people may experience challenges related to fertility, relationships, and genetic predisposition and support needs to enable caring for children [26]**.** Clinicians were limited to a few types of allied health and medical professionals. Their views may therefore not reflect the views of all healthcare professionals and CRC patients in all care settings. Specialists and support services are often outside of cancer such as physiotherapy to help with bowel function or podiatry to manage foot reactions related to chemotherapy (e.g. capecitabine induced palmar plantar erythrodysesthesia). Furthermore, we were not able to explore any systemic differences in experiences between genders as our research question did not include aims specific to this. Future research may help to elicit broader perspectives by sampling a more diverse cohort in terms of age, gender, and clinician type. The findings do however highlight areas where improvements to survivorship care could be made. We also did not specifically ask about use of survivorship care plans, although some participants mentioned these would be beneficial. The Cancer Council Australia Optimal Care Pathway for people with CRC states that screening and referral to supportive care should be considered throughout all cancer care and patients provided with access to multidisciplinary support services, groups, and therapies to meet their needs and optimise recovery [27]. Better uptake of the current version of the pathway might address some of the gaps highlighted in this study.

Given the growing population of CRC survivors experiencing consequences of treatment, detecting and managing these is crucial in posttreatment survivorship to improve patient outcomes and HRQL. Key to achieving this is more focused assessment, individualised care planning involving relevant healthcare professionals, access to the range of available supports in the community when needed, and improved information provision.

## Data Availability

The data that support the findings of this study are available from the corresponding author [CR] upon request.
